# Concurrent Validity of the Ergotex Device for Measuring Low Back Posture

**DOI:** 10.3390/bioengineering11010098

**Published:** 2024-01-20

**Authors:** Marco A. García-Luna, Jose M. Jimenez-Olmedo, Basilio Pueo, Carmen Manchado, Juan M. Cortell-Tormo

**Affiliations:** 1 Health, Physical Activity, and Sports Technology Research Group, Faculty of Education, University of Alicante, 03690 San Vicente del Raspeig, Spain; marco.garcia@ua.es (M.A.G.-L.); basilio@ua.es (B.P.), jm.cortell@ua.es (J.M.C.-T.); 2 Sports Coaching and Performance Research Group, Faculty of Education, University of Alicante, 03690 San Vicente del Raspeig, Spain; carmen.manchado@ua.es

**Keywords:** kinematics, IMU, wearable, instrumentation, posture

## Abstract

Highlighting the crucial role of monitoring and quantifying lumbopelvic rhythm for spinal curvature, the Ergotex IMU, a portable, lightweight, cost-effective, and energy-efficient technology, has been specifically designed for the pelvic and lumbar area. This study investigates the concurrent validity of the Ergotex device in measuring sagittal pelvic tilt angle. We utilized an observational, repeated measures design with healthy adult males (mean age: 39.3 ± 7.6 y, body mass: 82.2 ± 13.0 kg, body height: 179 ± 8 cm), comparing Ergotex with a 3D optical tracking system. Participants performed pelvic tilt movements in anterior, neutral, and posterior conditions. Statistical analysis included paired samples *t*-tests, Bland–Altman plots, and regression analysis. The findings show minimal systematic error (0.08° overall) and high agreement between the Ergotex and optical tracking, with most data points falling within limits of agreement of Bland–Altman plots (around ±2°). Significant differences were observed only in the anterior condition (0.35°, *p* < 0.05), with trivial effect sizes (ES = 0.08), indicating that these differences may not be clinically meaningful. The high Pearson’s correlation coefficients across conditions underscore a robust linear relationship between devices (*r* > 0.9 for all conditions). Regression analysis showed a standard error of estimate (SEE) of 1.1° with small effect (standardized SEE < 0.26 for all conditions), meaning that the expected average deviation from the true value is around 1°. These findings validate the Ergotex as an effective, portable, and cost-efficient tool for assessing sagittal pelvic tilt, with practical implications in clinical and sports settings where traditional methods might be impractical or costly.

## 1. Introduction

The lumbar lordosis is often one of the most relevant parameters in the study of the sagittal plane of the spine, and its normal range can vary in each individual, generally in relation to sacral slope or pelvic incidence [1,2]. Additionally, the sagittal curvatures of the vertebral column (i.e., lumbar and cervical lordosis, and thoracic and sacral kyphosis) have a relationship of interdependence [3,4]. In this regard, the thoracic curve is dependent on the orientation of the lumbar lordosis and vice versa, just as lumbar lordosis mainly depends on pelvic and sacral inclination [3,4,5,6]. Thus, pelvic movement in the sagittal plane (especially anterior and posterior pelvic tilt) directly affects the movement of the lumbar spine in the same plane (i.e., increasing and decreasing lordosis, respectively) [7,8,9,10].

The quantification and evaluation of spinal curves became a key factor in the medical field many decades ago [11]. Techniques for analyzing spinal movement have evolved significantly in recent decades, including 3D optical tracking systems and electromagnetic tracking devices, among others [12,13,14,15,16,17,18,19,20]. Generally, techniques such as photogrammetry, inertial/magnetic systems, electronic goniometers, and/or extensometer gauges have been used [21,22]. In this sense, the gold standard method for evaluating pelvic tilt is through radiographic measurements, although its primary drawbacks include radiation exposure and high costs [23]. Also, 3D optical motion capture systems have been extensively employed for assessing human motion, yet their primary drawback is the requirement for expensive laboratory equipment and setup, as well as a controlled environment [24]. The use of goniometers has also been widely adopted traditionally, though its main limitation lies in the inherent involvement of potential human error in measurement [25]. In summary, while these methods are capable of recording a large amount of precise and detailed data in most cases, their main limitations lie in their cost, the need for expertise in handling them, or the lack of portability (e.g., requiring controlled environments or laboratories specifically equipped for this purpose) [21,22].

Thanks to technological advances and with the aim of addressing these issues, wearable systems emerged [26]. They are capable of avoiding the risk of radiography radiation, are capable of providing quantitative data unlike traditional visual observation, and seem to offer greater validity and lower chances of measurement error during data collection compared with tools such as, for example, flexicurve [27,28]. The main advantages of these noninvasive systems are their portability, lightness, low cost, and energy efficiency [26,29,30]. Various wearable instruments are available for analyzing spinal movement [24,31,32,33], although inertial measurement units (IMUs) are often the most widely used [24,31]. Typically, IMUs are composed of one or more accelerometers and/or gyroscopes, and some may also include one or more magnetometers (to calibrate the sensors with reference to the Earth’s magnetic field and prevent drift bias) [31]. Through the measurement of angular velocity, IMUs can adequately estimate, among other variables, sensor inclination or joint angulation [28,34,35].

The use of inertial technology through IMUs to measure and quantify pelvic orientation is relatively new. Measuring pelvic tilt angle directly through IMUs in both static and dynamic positions has the potential to enhance angle measurement accuracy, offering numerical data compared with traditional methods like palpation or observation [36]. Employing IMUs for recording pelvic tilt could serve as a valuable tool for clinicians to monitor patients and offer improved feedback on the quality of movement more precisely. However, a potential limitation of these devices is the necessity for careful fixation to the person’s pelvis rather than the back, ensuring they accurately track pelvic movement rather than trunk movement [37].

Several studies have used inertial sensors to analyze pelvic movement in different planes and/or in different movements/contexts [38,39,40,41,42,43,44]. Some of them have been used as an evaluation method in total hip arthroplasty patients [41,42] or patients with reduced lumbar lordosis (or flat back) posture [40]. Others have analyzed their validity compared with 3D optical tracking systems during walking and sit–stand and step-up transfers [38,44] or during trunk flexion–extension movements [39]. They have also been used to investigate differences between sexes and speed levels in pelvic kinematics during running [43]. Also, studies have shown that wearable motion sensors and mobile devices have opened new possibilities in telerehabilitation, especially for balance and gait disorders. Systematic reviews have found improvements in balance and gait performance following long-term use of smartphone- and tablet-based rehabilitation technology [45].

However, the main limitations that IMUs face when quantifying the movement and disposition of the pelvis and spine can be summarized in two points [24,32]. The first point is the size of the devices. In situations where it is necessary to record the individual movement of adjacent vertebrae (i.e., placing a sensor on each of them), the larger size of the devices leads to them coming into contact and colliding with each other. This can occur mainly during anterior pelvic tilt and/or spine extension movements, adding excessive noise to the data recorded. Additionally, in subjects with highly developed involved musculature (e.g., quadratus lumborum, spinal erectors), it is necessary for the devices to be as compact as possible for proper placement on the vertebral processes. Second, the typical method of attaching sensors to the target anatomical structure is through adjustable straps or suits with pockets in generic locations. These types of attachments can cause slack or unintended movements of the sensors, further contributing to noise in the data and reducing their practical utility.

The IMU Ergotex evolved from the Lumbatex project, which involved designing a system of IMUs integrated into a textile garment to monitor curvature and spinal motion [46]. After this pioneering project in the monitoring and quantification of lumbar vertebral disposition [46], efforts were focused on the development of a new, more reliable and accurate device (i.e., Ergotex IMU) that would reduce noise caused by the textile garment. With this goal in mind, the decision was made to eliminate the textile garment and create an encapsulation as small and compact as possible. Additionally, attention was given to enhancing fixation using double-sided tape, and square prototypes of Kinesiotape with small perforations in their central part were created.

Thus, Ergotex is a smartphone- or tablet-based technology and is specifically applicable to the analysis of lumbar or pelvic kinematics in static (e.g., balance, stability) or dynamic (e.g., gait or squatting patterns) conditions. This integration aligns with the trend in rehabilitation medicine towards using mobile health (mHealth) applications, which have shown a breadth of applications in various rehabilitation contexts [47]. The manufacturer’s specifications indicate that the IMU Ergotex is capable of monitoring lumbar curvature and pelvic inclination with various sensors, but to date, there is no validation study that has assessed the accuracy of this device. Therefore, the aim of this study was to quantify the concurrent validity of Ergotex in measuring the sagittal pelvic tilt angle in healthy adults, compared with a 3D motion capture system considered the gold standard when analyzing human movement kinematics.

## 2. Materials and Methods

### 2.1. Study Design

This observational study aimed to ascertain concurrent validity through a repeated measures design, measuring sagittal pelvic tilt angle across three conditions: anterior pelvic tilt, neutral, and posterior pelvic tilt. Data were concurrently collected from two distinct devices to compare the readings acquired by the Ergotex IMU with those of a laboratory optical tracking system, serving as the criterion instrument. We adopted a within-subjects design to minimize variability and enhance the sensitivity of our comparison between devices. The Ergotex IMU was placed on the participant’s sacrum, ensuring accurate measurement of pelvic tilt. Calibration procedures were followed as per the manufacturer’s instructions to ensure the accuracy of both Ergotex IMU and the optical tracking system before each testing session. For a statistical power of 95%, a minimum of 45 concurrent measurements was determined using G*Power (v3.1.9.7, Heinrich-Heine-Universität Düsseldorf, Düsseldorf, Germany) with an alpha level of 0.1 for a two-tailed test. To achieve this number of measurements in each condition (anterior, neutral, and posterior pelvic tilt), the 9 participants performed 5 complete pelvic tilt transitions. Each transition was carefully monitored to maintain consistent movement speeds across trials. Additionally, we incorporated rest periods between each set of movements to reduce the risk of fatigue influencing the measurements. The selection of healthy male adults as participants was a deliberate choice aimed at establishing a clear baseline for the Ergotex device. By limiting our study to this group, variables such as gender-specific anatomical differences and potential variations in pelvic tilt mechanics can be controlled. It was also ensured that all participants were free from any known musculoskeletal disorders, as confirmed by a pre-study screening questionnaire. This approach was crucial in ensuring the reliability of our baseline measurements against the optical tracking system.

### 2.2. Instruments

#### 2.2.1. Ergotex

**Figure 1:**
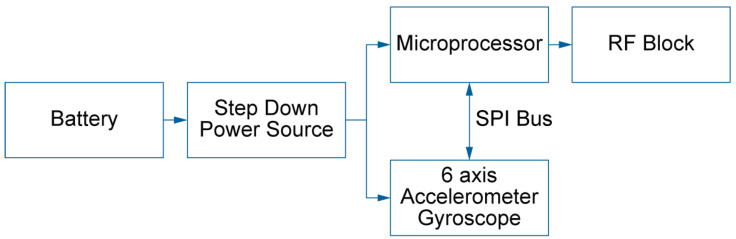
Working schematic of the IMU Ergotex.

The IMU Ergotex (JVTech Solutions, Alicante, Spain) comprises a 3-axis gyroscope (±1000 deg/s) and a 3-axis accelerometer (±2 g), encapsulated in a device of dimensions 23 × 21 × 10 mm and weight 8 g. The ICM-20602 MEMS MotionTracking (TDK Corp., Tokyo, Japan) device was selected for its high-performance specifications, critical for the reliability of the device. The gyroscope’s sensitivity error is within ±1%, and its noise level is low at ±4 mdeg/s/√Hz. Similarly, the accelerometer noise is tightly controlled at 100 µg/√Hz. The integration of a 1K-byte FIFO buffer minimizes serial bus traffic, thereby improving both the repeatability of measurements and the device’s power management. These features ensure consistent response times and sensitivity levels, which are essential for the accuracy of the experimental data. Figure 1 presents the working schematic of our device, designed for optimal energy efficiency and data integrity. The system is powered by a battery connected to a step-down power source, ensuring a stable voltage supply. The microprocessor forms the central unit, managing data from the 6-axis accelerometer and gyroscope via the SPI bus and transmitting processed information to the RF block for communication. This design ensures a seamless flow of data, vital for the accuracy and reliability of the device’s performance. The chosen ICM-20602 sensor provides a compact and energy-efficient solution for motion tracking, ideal for the targeted application in our study. Its small footprint and integrated FIFO buffer for data management offer significant advantages in terms of device miniaturization and power savings.

The IMU Ergotex operates at a sampling rate of 20 Hz and can be affixed to the skin using double-sided tape or secured elsewhere using an elastic strap. Primarily designed for monitoring spine posture, this device records acceleration data across all three axes. Internal integration of the acceleration signal occurs within the device, transmitting data instantaneously via Bluetooth (frequency: 2.4 GHz) to a smartphone or tablet equipped with the preinstalled Ergotex app. This application enables immediate data visualization and facilitates exportation to a spreadsheet in comma-separated text (CSV) format.

Prior to each measurement, the device undergoes calibration on all axes. This calibration process involves not only placing the device on a completely flat surface, but also ensuring it is in a stable, vibration-free environment to accurately establish the local three-dimensional coordinates of the IMU. This allows sufficient time for the software to recognize all six faces of the octahedron and establish the local three-dimensional coordinates of the IMU. Once calibrated, the device is activated 5 min before each test session and before subjects are instrumented. In addition to the standard calibration, we address the potential drifting issue, a known concern in IMU devices. Preceding the testing phase, a static trial is conducted by placing the device on the ground for approximately 10 s. These measurements are utilized to estimate and subtract the gyroscope bias [48]. To further mitigate gyroscope drift, the ‘zero-rate’ calibration is performed during this static trial, establishing a baseline for any potential drift. This step ensures higher accuracy by compensating for potential drifts detected during the static and dynamic phases of the experiment.

#### 2.2.2. Optical Tracking System OptiTrack

**Figure 2:**
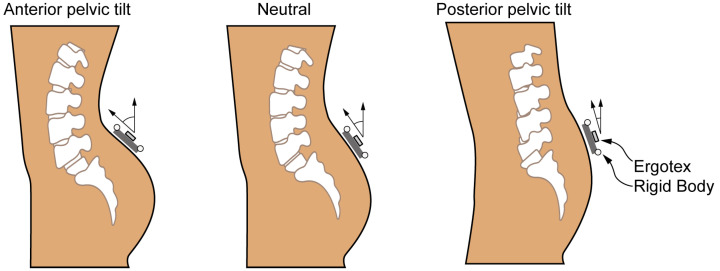
Experimental setup showing the three conditions under test.

The OptiTrack optical tracking system (NaturalPoint Inc., Corvallis, OR, USA) enables real-time determination of both position and orientation (six degrees of freedom) for rigid bodies. These entities are defined as structures equipped with a minimum of three optical markers, ensuring that distances between any two points and angles between any two vectors of the rigid body remain constant over time. Passive spheres with retroreflective coatings serve as optical markers, reflecting received infrared light in the same direction. Around the capture area, eight synchronized OptiTrack FLEX:V100R2 cameras (NaturalPoint Inc., Corvallis, OR, USA) with a resolution of 640 × 480 pixels (VGA) and a maximum frame rate of 100 fps were strategically positioned for this study. Each camera emits infrared light using 26 surrounding LEDs (IR 850 nm) in a ring configuration, synchronized with the capture shutter. Operating at 100 Hz with a shutter speed of 20 µs, these cameras provide an overall resolution of 0.001 m. This multicamera setup covers a substantial working volume, making it highly suitable for the intended procedure. The Tracking Tools software (v. 2.5.3, NaturalPoint Inc., Corvallis, OR, USA) processes the information captured by the near-infrared cameras to precisely determine the pose of any rigid body within the physical space. Extrinsic camera parameters (physical position and orientation) and intrinsic camera parameters (focal length and lens distortion) underwent calibration. This calibration procedure involved using a three-marker OptiWand (NaturalPoint Inc., Corvallis, OR, USA) along with the three-marker calibration algorithm within the Tracking Tools software [49,50]. The data were transmitted via a USB cable connection to a laptop, stored, and analyzed using the Tracking Tools software. This software not only synchronizes and calibrates the image collection systems but also exports the obtained data in CSV format for subsequent spreadsheet analysis, thereby determining the orientation of the rigid body. To establish the gold standard orientation of the Ergotex, a rigid body with four spherical optical markers (diameter 1.1 cm, Hand Rigid Body, NaturalPoint Inc.) was affixed to the Ergotex sensor. The markers were arranged coplanarly around the sensor, aiding in determining the gold standard orientation. This methodology was adopted as the gold standard due to the precision in marker position measurements achieved through an optoelectronic camera system [35]. The markers were precisely placed on rigid plastic boards, effectively minimizing skin movement artifacts [51]. The Ergotex and the optical rigid body, attached to the pelvic segment, were positioned atop the sacrum [52], as shown in Figure 2.

### 2.3. Procedure

The experimental procedure took place in a single session at the Movement Analysis laboratory of the University of Alicante. Prior to commencing the measurements, participants underwent familiarization with the experimental protocols, and anthropometric measurements were conducted. Subsequently, a researcher experienced in selecting anatomical landmarks affixed the optical rigid body and the Ergotex to the lower back section at the S1 position on the sacrum using double-sided tape.

Participants were then instructed to execute the necessary movements to achieve three conditions—anterior pelvic tilt, neutral, and posterior pelvic tilt—in a natural and steady manner. Each condition was maintained during 3 s to ensure that participants could comfortably attain and hold each condition in a natural and steady manner, allowing for precise and consistent data collection. After becoming acquainted with the protocol, each participant underwent 5 complete pelvic tilt transitions in a standing position. Each transition included the following sequence: anterior to neutral, neutral to posterior, and posterior back to neutral. This sequence was repeated four times, with the fifth and final transition concluding in the posterior position. Thus, each subject performed 5 instances of both anterior and posterior positions, and 9 instances of the neutral position, resulting in a total of 45 measurements for both anterior and posterior positions and 81 measurements for the neutral position across the 9 subjects. For each position, measurements were concurrently taken with both measuring instruments—the Ergotex and the optical tracking system.

### 2.4. Participants

Nine healthy male adults participated in this study (39.3 ± 7.6 y, body mass 82.2 ± 13.0 kg, body height 179 ± 8 cm). The inclusion criteria were as follows: absence of any known musculoskeletal or neurological disorders affecting the lower back or pelvic region, no history of recent surgeries or injuries to the lower back or pelvic area within the past six months, ability to understand and perform the instructed pelvic tilt movements as required during the experiment. The study was carried out in accordance with the guidelines of the ethical principles of the Declaration of Helsinki. All subjects provided informed written consent before the beginning of this study, which was approved by the Ethics Committee of the University of Alicante (protocol code UA-2023-11-16).

### 2.5. Statistical Analysis

Descriptive statistics are presented as mean ± SD (standard deviation), and 95% confidence intervals (95% CI). To examine systematic error, significant differences between the Ergotex and the criterion instrument were evaluated using a paired samples *t*-test. The effect size (ES) was computed as the bias-corrected Hedges ES [53], with interpretation of differences expressed as *g* according to Hopkins et al. [53]: trivial (<0.2), small (0.2–0.6), moderate (0.6–1.2), and large (>1.2). Bland–Altman plots were used to explore agreement between the instruments [54], which show mean outcome pairs against their difference between values to identify any random error and proportional bias. Proportional error was identified if the slope of the regression line and the Pearson’s correlation coefficient (*r*) of the differences against the mean differ significantly from zero (*p* > 0.05) [55]. The relationship between instruments was further analyzed using the bivariate Pearson’s product-moment correlation coefficient (*r*) with 95% CI. Thresholds for interpretation were set as follows: trivial (<0.1), small (0.1–0.3), moderate (0.3–0.5), high (0.5–0.7), very high (0.7–0.9), and practically perfect (>0.9) [56]. Regression analysis was employed to assess the linear relationship between paired data from both instruments, represented by the equation *y* = *ax* + *b*. This equation facilitates the prediction of the dependent variable *y* based on the independent variable *x*. The slope *a*, ideally set at 1, provides insights into the proportional differences between the two methods. Meanwhile, the intercept *b* at the *x*-axis, ideally null, delineates the systematic disparities between the two devices in a quantitative context. The standard error of estimate (SEE), computed in raw and standardized units [57], was interpreted using thresholds adapted from Cohen’s scale: trivial (<0.1), small (0.1–0.3), moderate (0.3–0.6), large (0.6–1.0), very large (1.0–2.0), and extremely large (>2.0) [56]. Lower SEE values indicate reduced error in estimation, suggesting closer alignment with the regression line. All statistical analyses were computed with IBM SPSS v. 22 (IBM Corp., Armonk, NY, USA) and an available spreadsheet for validity [58].

## 3. Results

Table 1 displays the mean differences observed between the measurements obtained from the Ergotex and optical tracking devices across different conditions. The mean differences ranged from 0.35 degrees for anterior pelvic tilt to −0.01 degrees for posterior pelvic tilt. Statistical analysis indicated no significant differences between the devices in the neutral (*p* = 0.881), posterior (*p* = 0.960), and overall conditions (*p* = 0.309). However, significant differences were observed in the anterior condition (*p* = 0.014).

**Figure 3:**
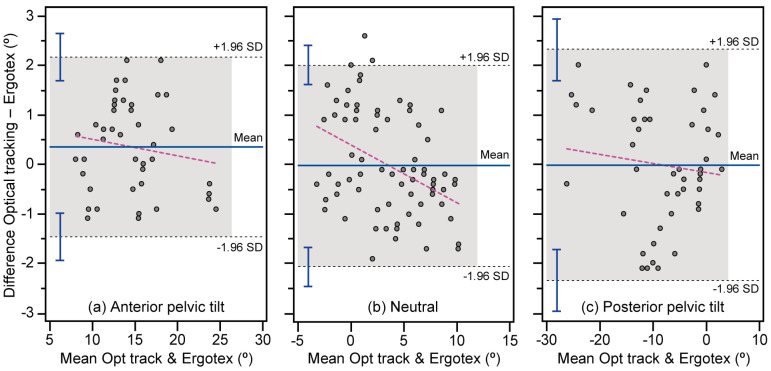
Agreement between angle measurements obtained from optical tracking and Ergotex for anterior (**a**), neutral (**b**), and posterior (**c**) conditions. The solid central line shows the mean between devices, representing the systematic bias, while the upper and lower dotted lines show mean ± 1.96 SD, as a measure of random error. The 95% confidence intervals (CI) are represented by bars. Additionally, the dashed line illustrates the linear regression of the differences and the mean, providing insights into any proportional bias.

The Bland–Altman analysis (Table 2) indicates low systematic error values and 95% CI, suggesting that the Ergotex provides estimations less than one degree compared with optical tracking. This implies a consistent tendency of the Ergotex to yield slightly lower values than optical tracking across the measured conditions. It is noteworthy that almost all data points fall within the LoA, as depicted in Figure 3. This indicates a high level of agreement between devices, considering the low dispersion of differences. The linear regression analysis of these differences, plotted against the mean, reveals slight deviations from homoscedasticity for both anterior and posterior conditions, as the slopes and Pearson product-moment correlation coefficients are indistinguishable from zero (*p* > 0.05). The neutral condition displayed heteroscedasticity, indicating that the Ergotex tends to yield lower values than optical tracking as sagittal pelvic tilt angles increase. This suggests a variance in the differences between devices concerning this specific condition. However, in the overall condition, there is almost a negligible slope (−0.001) and an *r* of −0.01, signifying that the Ergotex does not contribute significantly to proportional error across the entirety of the measurements.

**Figure 4:**
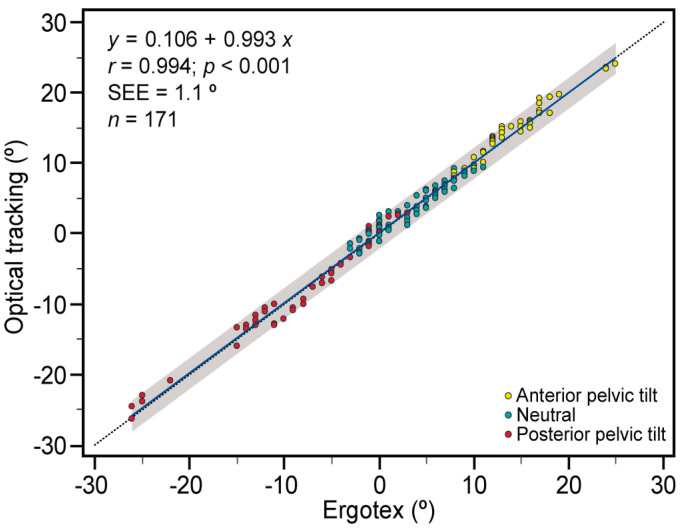
Relationship between angle measurements obtained from optical tracking and Ergotex for anterior (yellow), neutral (blue), and posterior (red) conditions. The solid blue line represents the linear regression, providing a trendline for the data. The dotted line indicates the line of equality (*y* = *x*) for reference. The gray shaded area represents the 95% prediction interval, offering insights into the expected range of measurements. SEE: standard error of the estimate, *r*: Pearson’s correlation coefficient. Each data point signifies a concurrent measurement with both devices.

The agreement between the two devices was further analyzed using linear regression analysis (Figure 4) across the three conditions. All data points fell within the 95% prediction interval, depicted as the gray shaded area, suggesting a high level of confidence in the predictions made by the linear regression model.

Table 3 demonstrates that the bivariate Pearson’s product-moment correlation coefficient (*r*) is practically perfect, exceeding 0.9 for all cases and within the entire 95% CI with a significance level of *p* < 0.001, indicating a robust association between their outputs. Moreover, the regression analysis provided equations for the fitted line, indicating that the Ergotex is capable of predicting outcomes with low error. The intercepts range from 0.11 to 0.84 degrees, indicating the starting point of the predicted values, while the slopes range from −0.02 to −0.12 degrees, representing the proportional relationship between the devices. The SEE was consistently low, around one degree for all conditions, assessed as small via the standardized SEE. This highlights minimal deviation of the observed values from the predicted values, suggesting a high degree of precision and accuracy in the predictions made by both devices across various conditions.

## 4. Discussion

This study aimed to analyze the concurrent validity of the IMU Ergotex when compared with a criterion instrument considered as the gold standard based on a 3D optical tracking system for the quantification of the sagittal pelvic tilt angle in healthy adults. The main results of this work show a high level of validity for the Ergotex when compared with optical tracking in estimating the sagittal inclination of the pelvis. This validity is notably bolstered by the minimal systematic error of 0.08°, affirming the Ergotex’s accuracy in pelvic tilt measurements. The implementation of this technology could assist clinicians and sports specialists in quantifying and monitoring the sagittal tilt of the pelvis and its consequent relationship with the lumbar spine in real time. The advantages that this technology presents over traditionally used methods could, in addition to reducing costs, broaden the possibilities for working with patients/athletes (e.g., in more realistic, less controlled, more versatile environments, etc.).

Comparing the Ergotex and optical tracking devices revealed nonsignificant differences in the neutral, posterior pelvic tilt, and overall conditions, suggesting comparable measurements between the devices in those scenarios. However, a statistically significant difference was detected in the anterior pelvic tilt condition, indicating divergent measurements during anterior movements. Despite the significant difference observed in the anterior condition, it is noteworthy that the effect size (ES) for all conditions, along with the limits of the 95% confidence interval (CI), were trivial. This suggests that while a statistically significant difference was found in the anterior condition, the practical significance or the magnitude of this difference might not be substantial or clinically meaningful.

In order to understand if the systematic error caused by integration is negligible or significant over time, it was found that the error range in the end stages of the pelvic tilt sequence did not differ substantially from the earlier stages. This finding suggests that the systematic error caused by integration in the IMU devices, particularly over the course of the pelvic tilt sequence, is minimal and does not significantly impact the overall accuracy of the measurements. Therefore, we conclude that the systematic error inherent to the IMU technology does not compromise the validity of our findings.

Although there are not many studies that have analyzed the sagittal movement of the pelvis using inertial technology in a more or less isolated manner, our results do not differ significantly from those found in similar studies [38,39,44]. This consistency across studies further reinforces the accuracy of the Ergotex in measuring pelvic tilt. In general, the majority of authors have not found significant differences between the IMU and the optical tracking system in the quantification of sagittal pelvic tilt [38,39]. Buganè et al. [44] reported certain differences at specific points in pelvic movement, but only in two out of nine comparisons. The differences (*p* < 0.05) were found in rotation and obliquity movements at specific walking speeds, and not in pelvic tilt [44].

The difference between the upper and lower LoAs, computed as ± 1.96 SD, ranges around ±1.8 degrees for anterior pelvic tilt and around ±2.1 degrees for the remaining conditions. Almost all data points fall within these LoAs, illustrating a high level of agreement between devices. This suggests a narrow dispersion of differences, indicating strong concordance between measurements from both devices. Our results align with previous studies in which Bland–Altman plots were also reported, showing the vast majority of points (except for isolated outliers under specific conditions) falling within two standard deviations (SDs) of error [38,39]. The neutral condition shows heteroscedasticity, wherein the Ergotex tends to provide lower values than the optical tracking with increasing sagittal pelvic tilt angles. However, the overall condition demonstrates a practically zero slope (−0.001) and a negligible *r* (−0.01), suggesting that the Ergotex does not introduce significant proportional error to the overall measurements.

The examination of data points within the 95% prediction interval reveals a robust predictive relationship between the devices, signifying a high level of certainty in the accuracy of predictions derived from the linear regression model. This comprehensive analysis, supported by the bivariate Pearson’s product-moment correlation coefficient (r) exceeding 0.9 for all cases within the entire 95% confidence interval (CI) at a significance level of *p* < 0.001, underlines an extremely strong linear relationship between the measurements from both devices. This robust association between their outputs stands as evidence of a solid connection, significantly beyond the realm of chance.

These results are consistent with the correlation values found in other studies [38,44]. Buganè et al. [44] reported an average correlation coefficient (*r*) of 0.90 for the movements of tilt, obliquity, and rotation of the pelvis, and 0.88 for the specific movement of sagittal pelvic tilt during the gait pattern. Similarly, Bolink et al. [38] found Pearson’s correlation coefficients (*r*) of 0.92 and 0.89 in pelvic movement in the frontal plane (i.e., obliquity) and sagittal plane (i.e., pelvic tilt), respectively, during walking and sit–stand and step-up transfers.

Moreover, the regression analysis equations, delineating the Ergotex’s predictive outcomes, showcase minimal error in its estimations. These equations, represented by intercepts ranging from 0.11 to 1.13 degrees and slopes from 0.86 to 0.99, highlight the accuracy and proportionality of the Ergotex’s predictions. The consistency observed in these values, closely aligning with the measurements of the optical tracking device across various conditions, underscores the reliability of the Ergotex’s predictions. Most studies of similar nature have not conducted regression analyses [38,39], making it challenging to compare our results with the existing literature. In the work of Buganè et al. [39] they performed a regression analysis to represent the differences in measurement between the two instruments in pelvic movement in the sagittal (tilt), frontal (obliquity), and transverse (rotation) planes. Through a linear regression model for the three planes of pelvic movement, they found good coefficients of determination (approximately 0.9 in most subjects) between the two instruments for obliquity and rotation, although it was poor for pelvic tilt [44].

Adding to the validation, the standard error of the estimate (SEE) persistently hovers around one degree for all conditions. This minimal deviation of observed values from predicted values serves as a testament to the high degree of precision and accuracy in the predictions made by both devices across a spectrum of conditions. These collective findings validate the reliability and accuracy of the Ergotex’s predictions while affirming the strong and consistent relationship between the measurements obtained from both devices. Buganè et al. [44] found very small errors (less than 1.5°) in obliquity and rotation movements, although they reported a considerable bias of about 7–8° for sagittal pelvic tilt [44]. However, the authors concluded that this error was likely due to the different angles of inclination in the sagittal plane measured by the two systems during the subject’s initial position and not to the instrument or the movements themselves [44].

Finally, to the best of the authors’ knowledge, this is the first study aiming to validate the IMU Ergotex for quantifying sagittal pelvic movement in healthy adults. It is unknown whether the promising results reported in this study are applicable to other pelvic movements or anatomical structures. In addition, it is important to emphasize that, regardless of the adequate results that this and other works are reporting on IMUs as tools for capturing human motion, this technology has not yet been implemented on a large scale. Therefore, it is essential to be aware that further investigation is needed into the reasons why its use is not widespread despite its advantages as a motion capture system, especially in uncontrolled real-world contexts.

Future research endeavors are needed to advance the understanding and quality of this device so that its use can be extended to other domains validly and reliably. Due to the satisfactory results found, it is suggested to focus on expanding the range of potential possibilities for this device by further studying it in different and/or more dynamic settings. For instance, it would be interesting to delve into the study and real-time quantification of pelvic tilt during the squat exercise to determine its healthy depth and potential associated factors (e.g., low back pain). It would probably also be helpful to use this technology as an educational tool for postural hygiene and control, fostering the learning of children and adolescents.

## 5. Conclusions

This study elucidates a high level of validity between the Ergotex and optical tracking devices in estimating spinal parameters across various conditions. The findings revealed consistent and precise predictions by the Ergotex, showcasing minimal error and strong proportional alignment with the measurements derived from the optical tracking device. Notably, the agreement within the 95% prediction interval, supported by a high bivariate Pearson’s product-moment correlation coefficient exceeding 0.9 across all cases, underscores the robustness and reliability of the Ergotex’s estimations.

Moreover, the negligible deviation observed via the SEE further substantiates the accuracy and precision of both devices’ measurements. These outcomes collectively validate the Ergotex’s capability to provide reliable estimations of spinal parameters.

This study’s findings hold significant implications for clinical applications, suggesting the potential for the Ergotex to serve as a reliable alternative to optical tracking in estimating spinal parameters. However, further research encompassing larger sample sizes and diverse demographic groups would fortify these findings and enhance the generalizability of the Ergotex’s applicability in clinical settings.

## Figures and Tables

**Table 1:** Descriptive statistics and mean differences in optical tracking and Ergotex sagittal pelvic tilt angle measurements (mean ± SD).

	Optical Tracking	Ergotex	Mean Diff	ES
Anterior pelvic tilt	15.06 ± 4.33	14.71 ± 4.47	0.35 ± 0.92 *	0.08
+95% CI	16.36	16.06	0.64	0.14
−95% CI	13.76	13.36	0.07	−0.03
Neutral	3.65 ± 3.53	3.67 ± 3.96	−0.02 ± 1.04	0.00
+95% CI	4.43	4.54	0.21	0.03
−95% CI	2.87	2.79	−0.25	−0.04
Posterior pelvic tilt	−8.26 ± 7.65	−8.27 ± 7.78	−0.01 ± 1.19	0.00
+95% CI	−5.98	−5.93	0.35	0.06
−95% CI	−10.57	−10.61	−0.37	−0.06
Overall	3.51 ± 9.89	3.43 ± 9.90	0.08 ± 1.05	0.01
+95% CI	5.00	4.92	0.24	0.02
−95% CI	2.02	1.94	−0.08	−0.01

* *p* < 0.05. Optical tracking, Ergotex, and mean difference (diff) are expressed in degrees. ES: effect size, CI: confidence intervals.

**Table 2:** Comparison of bias, limits of agreement, interval estimates, and regression parameters for anterior, neutral, posterior, and overall conditions derived from Bland–Altman analysis.

	Bias	+LoA	−LoA	Intercept	Slope	*r*	*p*
Anterior pelvic tilt	0.35 *	2.17	−1.46	0.84	−0.04	−0.16	0.31
+95% CI	0.63	2.64	−0.98	1.84	0.03	0.14	
−95% CI	0.07	1.69	−1.94	−0.15	−0.09	−0.43	
Neutral	−0.02	2.01	−2.05	0.41	−0.12	−0.41	<0.001
+95% CI	0.21	2.41	−1.66	0.70	−0.06	−0.22	
−95% CI	−0.25	1.62	−2.44	0.11	−0.17	−0.58	
Posterior pelvic tilt	−0.01	2.32	−2.33	−0.16	−0.02	−0.12	0.44
+95% CI	0.35	2.94	−1.72	0.37	0.37	0.18	
−95% CI	−0.37	1.71	−2.95	−0.69	−0.69	−0.39	
Overall	0.08	2.16	−2.00	0.08	−0.001	−0.01	0.89
+95% CI	0.24	2.43	−1.72	0.26	0.015	0.14	
−95% CI	−0.08	1.89	−2.26	−0.08	−0.017	−0.16	

* *p* < 0.05. Bias, LoA (limit of agreement), and intercept are expressed in degrees. *r*: Pearson’s correlation coefficient of the differences, *p*: *p*-value of the slope, and *r* for heterosdacidity assessment; CI: confidence intervals.

**Table 3:** Regression line analysis for angle measurements: optical tracking vs. Ergotex.

	Intercept	Slope	*r*	SEE	Stdz. SEE
Anterior pelvic tilt	1.13	0.95	0.994 **	0.91	0.21 (S)
+95% CI	2.08	1.00	0.997	1.15	0.29 (S)
−95% CI	0.19	0.88	0.990	0.75	0.16 (S)
Neutral	0.49	0.86	0.968 **	0.89	0.26 (S)
+95% CI	0.75	0.91	0.979	1.06	0.33 (M)
−95% CI	0.21	0.81	0.951	0.77	0.21 (S)
Posterior pelvic tilt	−0.25	0.97	0.988 **	1.18	0.15 (S)
+95% CI	0.27	0.27	0.994	1.50	0.21 (S)
−95% CI	−0.77	−0.77	0.979	0.98	0.11 (S)
Overall	0.11	0.99	0.994 **	1.06	0.11 (S)
+95% CI	0.27	1.00	0.996	1.19	0.12 (S)
−95% CI	−0.06	0.97	0.992	0.96	0.09 (T)

** *p* < 0.001. Intercept and standard error of the estimate (SEE) are expressed in degrees. *r*: Pearson’s correlation coefficient, CI: confidence intervals, standardized (Stdz.) SEE assessment: trivial (T), small (S), moderate (M).

## Data Availability

The data that support the findings of this study are available from the corresponding author upon reasonable request.

## References

[1] Been E., Kalichman L. (2014). Lumbar lordosis. Spine J..

[2] Bailey J.F., Shefi S., Soudack M., Kramer P.A., Been E. (2019). Development of pelvic incidence and lumbar lordosis in children and adolescents. Anat. Rec..

[3] Roussouly P., Pinheiro-Franco J.L. (2011). Sagittal parameters of the spine: Biomechanical approach. Eur. Spine J..

[4] During J., Goudfrooij H., Keessen W., Beeker T.W., Crowe A. (1985). Toward standards for posture: Postural characteristics of the lower back system in normal and pathologic conditions. Spine.

[5] Le Huec J., Aunoble S., Philippe L., Nicolas P. (2011). Pelvic parameters: Origin and significance. Eur. Spine J..

[6] Legaye J., Duval-Beaupere G., Hecquet J., Marty C. (1998). Pelvic incidence: A fundamental pelvic parameter for three-dimensional regulation of spinal sagittal curves. Eur. Spine J..

[7] Vaz G., Roussouly P., Berthonnaud E., Dimnet J. (2002). Sagittal morphology and equilibrium of pelvis and spine. Eur. Spine J..

[8] Levine D., Whittle M.W. (1996). The effects of pelvic movement on lumbar lordosis in the standing position. J. Orthop. Sports Phys. Ther..

[9] Keegan J.J. (1953). Alterations of the lumbar curve related to posture and seating. J. Bone Jt. Surg..

[10] Day J.W., Smidt G.L., Lehmann T. (1984). Effect of pelvic tilt on standing posture. Phys. Ther..

[11] Vrtovec T., Pernuš F., Likar B. (2009). A review of methods for quantitative evaluation of spinal curvature. Eur. Spine J..

[12] Mannion A.F., Knecht K., Balaban G., Dvorak J., Grob D. (2004). A new skin-surface device for measuring the curvature and global and segmental ranges of motion of the spine: Reliability of measurements and comparison with data reviewed from the literature. Eur. Spine J..

[13] Schmid S., Studer D., Hasler C., Romkes J., Taylor W.R., Lorenzetti S., Brunner R. (2016). Quantifying spinal gait kinematics using an enhanced optical motion capture approach in adolescent idiopathic scoliosis. Gait Posture.

[14] Ranavolo A., Don R., Draicchio F., Bartolo M., Serrao M., Padua L., Cipolla G., Pierelli F., Iavicoli S., Sandrini G. (2013). Modelling the spine as a deformable body: Feasibility of reconstruction using an optoelectronic system. Appl. Ergon..

[15] Consmüller T., Rohlmann A., Weinland D., Druschel C., Duda G.N., Taylor W.R. (2012). Comparative evaluation of a novel measurement tool to assess lumbar spine posture and range of motion. Eur. Spine J..

[16] Taylor W.R., Consmüller T., Rohlmann A. (2010). A novel system for the dynamic assessment of back shape. Med. Eng. Phys..

[17] O’Sullivan K., O’Sullivan L., Campbell A., O’Sullivan P., Dankaerts W. (2012). Towards monitoring lumbo-pelvic posture in real-life situations: Concurrent validity of a novel posture monitor and a traditional laboratory-based motion analysis system. Man. Ther..

[18] Mieritz R.M., Bronfort G., Jakobsen M.D., Aagaard P., Hartvigsen J. (2014). Reliability and measurement error of sagittal spinal motion parameters in 220 patients with chronic low back pain using a three-dimensional measurement device. Spine J..

[19] Mitchell T., O’Sullivan P.B., Burnett A.F., Straker L., Smith A. (2008). Regional differences in lumbar spinal posture and the influence of low back pain. BMC Musculoskelet. Disord..

[20] Müller R., Ertelt T., Blickhan R. (2015). Low back pain affects trunk as well as lower limb movements during walking and running. J. Biomech..

[21] Rosário J.L.P.d. (2014). Biomechanical assessment of human posture: A literature review. J. Bodyw. Mov. Ther..

[22] Lehman G.J. (2004). Biomechanical assessments of lumbar spinal function. how low back pain sufferers differ from normals. implications for outcome measures research. Part I: Kinematic assessments of lumbar function. J. Manip. Physiol. Ther..

[23] Bierma-Zeinstra S.M., van Gool J.J., Bernsen R.M., Njoo K.H. (2001). Measuring the sacral inclination angle in clinical practice: Is there an alternative to radiographs?. J. Manip. Physiol. Ther..

[24] Papi E., Koh W.S., McGregor A.H. (2017). Wearable technology for spine movement assessment: A systematic review. J. Biomech..

[25] Mayer T.G., Kondraske G., Beals S.B., Gatchel R.J. (1997). Spinal range of motion: Accuracy and sources of error with in-clinometric measurement. Spine.

[26] Bonato P. (2010). Wearable sensors and systems. IEEE Eng. Med. Biol. Mag..

[27] de Oliveira T.S., Candotti C.T., La Torre M., Pelinson P.P.T., Furlanetto T.S., Kutchak F.M., Loss J.F. (2012). Validity and reproducibility of the measurements obtained using the flexicurve instrument to evaluate the angles of thoracic and lumbar curvatures of the spine in the sagittal plane. Rehabil. Res. Pract..

[28] Poitras I., Dupuis F., Bielmann M., Campeau-Lecours A., Mercier C., Bouyer L.J., Roy J. (2019). Validity and reliability of wearable sensors for joint angle estimation: A systematic review. Sensors.

[29] Shull P.B., Jirattigalachote W., Hunt M.A., Cutkosky M.R., Delp S.L. (2014). Quantified self and human movement: A review on the clinical impact of wearable sensing and feedback for gait analysis and intervention. Gait Posture.

[30] Fong D.T., Chan Y. (2010). The use of wearable inertial motion sensors in human lower limb biomechanics studies: A systematic review. Sensors.

[31] Simpson L., Maharaj M.M., Mobbs R.J. (2019). The role of wearables in spinal posture analysis: A systematic review. BMC Musculoskelet. Disord..

[32] Hodges P.W., van den Hoorn W. (2022). A vision for the future of wearable sensors in spine care and its challenges: Narrative review. J. Spine Surg..

[33] Vásquez-Ucho P.A., Villalba-Meneses G.F., Pila-Varela K.O., Villalba-Meneses C.P., Iglesias I., Almeida-Galárraga D.A. (2021). Analysis and evaluation of the systems used for the assessment of the cervical spine function: A systematic review. J. Med. Eng. Technol..

[34] Walmsley C.P., Williams S.A., Grisbrook T., Elliott C., Imms C., Campbell A. (2018). Measurement of upper limb range of motion using wearable sensors: A systematic review. Sports Med. Open.

[35] Cuesta-Vargas A.I., Galán-Mercant A., Williams J.M. (2010). The use of inertial sensors system for human motion analysis. Phys. Ther. Rev..

[36] Picerno P. (2017). 25 years of lower limb joint kinematics by using inertial and magnetic sensors: A review of methodolog-ical approaches. Gait Posture.

[37] Ismail I., Narayanan A.L.T., Wicaksono D.H.B. Comparison of two sagittal pelvic tilt measurement protocols using newly calibrated novel pelvic sensor. Proceedings of the 2011 2nd International Conference on Instrumentation Control and Automation.

[38] Bolink S.A.A.N., Naisas H., Senden R., Essers H., Heyligers I.C., Meijer K., Grimm B. (2016). Validity of an inertial measurement unit to assess pelvic orientation angles during gait, sit–stand transfers and step-up transfers: Comparison with an optoelectronic motion capture system*. Med. Eng. Phys..

[39] Beange K.H.E., Chan A.D.C., Beaudette S.M., Graham R.B. (2019). Concurrent validity of a wearable IMU for objective assessments of functional movement quality and control of the lumbar spine. J. Biomech..

[40] Shin S., Yoo W. (2019). Inertial Measurement unit-based evaluation of global and regional lumbar spine and pelvis alignment in standing individuals with a flat lumbar posture. J. Manip. Physiol. Ther..

[41] Wang X., Qureshi A., Vepa A., Rahman U., Palit A., Williams M.A., King R., Elliott M.T. (2020). A sensor-based screening tool for identifying high pelvic mobility in patients due to undergo total hip arthroplasty. Sensors.

[42] Vayalapra S., Wang X., Qureshi A., Vepa A., Rahman U., Palit A., Williams M.A., King R., Elliott M.T. (2022). Repeatability of inertial measurement units for measuring pelvic mobility in patients undergoing total hip arthroplasty. Sensors.

[43] Perpiñá-Martínez S., Arguisuelas-Martínez M.D., Pérez-Domínguez B., Nacher-Moltó I., Martínez-Gramage J. (2023). Differences between sexes and speed levels in pelvic 3D kinematic patterns during running using an inertial measurement unit (IMU). Int. J. Environ. Res. Public Health.

[44] Buganè F., Benedetti M.G., D’Angeli V., Leardini A. (2014). Estimation of pelvis kinematics in level walking based on a single inertial sensor positioned close to the sacrum: Validation on healthy subjects with stereophotogrammetric system. Biomed. Eng. Online.

[45] Lee C., Ahn J., Lee B. (2023). A systematic review of the long-term effects of using smartphone-and tablet-based rehabilitation technology for balance and gait training and exercise programs. Bioengineering.

[46] Cortell-Tormo J., Garcia-Jaen M., Ruiz-Fernandez D., Fuster-Lloret V. (2019). Lumbatex: A wearable monitoring system based on inertial sensors to measure and control the lumbar spine motion. IEEE Trans. Neural Syst. Rehabil. Eng. A Publ. IEEE Eng. Med. Biol. Soc..

[47] Ramey L., Osborne C., Kasitinon D., Juengst S. (2019). Apps and mobile health technology in rehabilitation: The good, the bad, and the unknown. Phys. Med. Rehabil. Clin. N. Am..

[48] Bergamini E., Ligorio G., Summa A., Vannozzi G., Cappozzo A., Sabatini A.M. (2014). Estimating orientation using magnetic and inertial sensors and different sensor fusion approaches: Accuracy assessment in manual and locomotion tasks. Sensors.

[49] Teufl W., Miezal M., Taetz B., Fröhlich M., Bleser G. (2019). Validity of inertial sensor based 3D joint kinematics of static and dynamic sport and physiotherapy specific movements. PLoS ONE.

[50] Brice S.M., Phillips E.J., Millett E.L., Hunter A., Philippa B. (2020). Comparing inertial measurement units and marker-based biomechanical models during dynamic rotation of the torso. Eur. J. Sport Sci..

[51] Cappozzo A., Catani F., Della Croce U., Leardini A. (1995). Position and orientation in space of bones during movement: Anatomical frame definition and determination. Clin. Biomech..

[52] Cutti A.G., Ferrari A., Garofalo P., Raggi M., Cappello A., Ferrari A. (2010). ‘Outwalk’: A protocol for clinical gait analysis based on inertial and magnetic sensors. Med. Biol. Eng. Comput..

[53] Hopkins W., Marshall S., Batterham A., Hanin J. (2009). Progressive statistics for studies in sports medicine and exercise science. Med. Sci. Sports Exerc..

[54] Bland J.M., Altman D.G. (1986). Statistical methods for assessing agreement between two methods of clinical measurement. Lancet.

[55] Ludbrook J. (2010). Confidence in Altman–Bland plots: A critical review of the method of differences. Clin. Exp. Pharmacol. Physiol..

[56] Hopkins W.G. (2018). Sportsci. Org. Sportscience.

[57] Pyne D. (2008). Measurement studies in sports science research. Int. J. Sports Physiol. Perform..

[58] Hopkins W.G. (2015). Spreadsheets for analysis of validity and reliability. Sportscience.

